# Baseline Hedgehog Pathway Activation and Increase of Plasma Wnt1 Protein Are Associated with Resistance to Immune Checkpoint Inhibitors in Advanced Non-Small-Cell Lung Cancer

**DOI:** 10.3390/cancers13051107

**Published:** 2021-03-05

**Authors:** Camille Mehlman, Paul Takam Kamga, Adrien Costantini, Catherine Julié, Coraline Dumenil, Jennifer Dumoulin, Julia Ouaknine, Violaine Giraud, Thierry Chinet, Jean-François Emile, Etienne Giroux Leprieur

**Affiliations:** 1 EA 4340 BECCOH, UVSQ, Université Paris-Saclay, 92100 Boulogne-Billancourt, France; camille.mehlman@aphp.fr (C.M.); takam.paul@gmail.com (P.T.K.); adrien.costantini@aphp.fr (A.C.); catherine.julie@aphp.fr (C.J.); thierry.chinet@aphp.fr (T.C.); jean-francois.emile@uvsq.fr (J.-F.E.); 2 Department of Respiratory Diseases and Thoracic Oncology, APHP—Hopital Ambroise Pare, 92100 Boulogne-Billancourt, France; coraline.dumenil@aphp.fr (C.D.); jennifer.dumoulin@aphp.fr (J.D.); julia.ouaknine@aphp.fr (J.O.); violaine.giraud@aphp.fr (V.G.); 3 Department of Pathology, APHP—Hopital Ambroise Pare, 92100 Boulogne-Billancourt, France

**Keywords:** non-small cell lung cancer, Sonic Hedgehog, Wnt, immune checkpoint inhibitor, resistance, Gli1, plasma, beta-catenin

## Abstract

**Simple Summary:**

Wnt and Hedgehog (Hh) pathways are associated with stemness profile and with resistance to anti-tumor therapies. Pre-clinical data have suggested a role of these pathways in resistance to immune checkpoint inhibitors (ICIs). Here we evaluated the expression and levels of Shh and Wnt molecules both in plasma and tumor samples in a prospective cohort of 63 consecutive NSCLC patients treated with ICIs. We found that baseline Hh activation as assessed by nuclear staining of Gli1 was associated with poor outcome and high primary resistance rate. Increase of plasma Wnt1 during treatment was associated with resistance to ICIs. This is the first study to our knowledge to find an association between the Hh and Wnt pathways and resistance to ICIs in advanced NSCLC patients. We believe that these results are important to understand the molecular mechanisms associated with resistance to ICIs, and ultimately to improve future treatment strategies.

**Abstract:**

Hedgehog (Hh) and Wingless-type (Wnt) pathways are associated with resistance to immune checkpoint inhibitors (ICIs) in preclinical studies. This study aimed to assess the association between expression and activation levels of Wnt and Sonic Hedgehog (Shh) pathways and resistance to ICIs in advanced NSCLC patients treated with ICI. Hh and Wnt pathways activation was assessed by immunohistochemistry (Gli1 and beta-catenin) on corresponding tumor tissues, and by plasma concentrations of Shh and Wnt (Wnt1, Wnt2 and Wnt3) at ICI introduction and at the first clinical evaluation. Sixty-three patients were included, with 36 patients (57.1%) with available tissue. Response rate was lower in Gli1+ NSCLC (20.0%) compared to Gli1 negative (Gli-) NSCLC (55.6%) (*p* = 0.015). Rate of primary resistance was 69.8%, vs. 31.2%, respectively (*p* = 0.04), and median progression-free survival (PFS) was 1.9 months (interquartile range (IQR) 1.2–5.7) vs. 6.1 months (1.6–26.0), respectively (*p* = 0.08). Median PFS and overall survival were shorter in case of increase of Wnt1 concentration during ICI treatment compared to other patients: 3.9 months vs. 11.2 months (*p* = 0.008), and 15.3 months vs. not reached (*p* = 0.003). In conclusion, baseline activation of Hh pathway and increase of Wnt1 concentrations during ICI treatment were associated with poor outcome in NSCLC patients treated with ICIs.

## 1. Introduction

Immune checkpoints inhibitors (ICIs) can restore anti-tumor immunity, notably by disrupting the immune checkpoint axis between cytotoxic T-lymphocytes and tumor cells, through the target of immune checkpoint molecules such as PD-1 (programmed death-1)/PD-L1 (programmed death-ligand 1) and cytotoxic T-lymphocyte-associated protein 4 (CTLA4). PD-1/PDL1-based-ICIs including nivolumab and pembrolizumab have demonstrated their efficacy in advanced non-small cell lung cancer (NSCLC), and are currently given in this setting in monotherapy (in first-line treatment in case of high expression of PD-L1 in immunohistochemistry (IHC) for pembrolizumab, or in second-line setting), or in combination with chemotherapy in first-line treatment [1,2,3,4]. 

Approximately 80% of patients treated in second line with ICIs and almost half of patients with high PD-L1 expression treated in first line with pembrolizumab monotherapy do not have tumor response, defining primary resistance. Moreover, around 20% of patients rapidly progress after introducing ICIs, defining a “hyperprogressive” pattern [5]. A large field of research on biomarkers associated with resistance to ICIs has emerged [6,7,8,9,10]. Emerging data support that cancer stem cells (CSCs) enhanced resistance to both chemotherapy and immunotherapy [11,12,13]. Indeed, stemness phenotype has also been described as associated with immune-resistance and ICI failure [14,15]. Persistence and survival of CSCs relay on the activation of developmental pathways including Wingless-type (Wnt) and Sonic Hedgehog (Shh)/Gli pathway [16]. Wnt signaling is activated when Wnt ligands bind to the receptors of the Frizzled family, leading to the stabilization of beta-catenin and its nuclear accumulation, that will trigger the expression of genes involved in cell survival and proliferation [17]. Wnt pathway is implicated in the maintenance of CSCs and bronchial carcinogenesis, and Wnt protein expression is associated with high tumor proliferation and poor prognosis in NSCLC [18,19,20]. Wnt pathway is also necessary for the formation, activation and/or suppression of immune cells such as cytotoxic CD8+ cells, regulatory T cells and B cells. Consistently, Wnt pathway activation were found associated with reduced antitumoral immunity. The latter is characterized by low peritumoral lymphocyte infiltration, especially in melanoma and hepatocarcinoma, and presumably with lower ICIs efficacy [21,22,23]. Molecular screening at the time of late progression on ICIs in advanced NSCLC has also shown the acquisition of molecular alterations in Wnt pathway-related genes, suggesting a role of this pathway in secondary resistance [24]. Lessons from developmental biology have established a crosstalk between Wnt signalling and Hedgehog (Hh) pathway. Hh pathway is activated when hedgehog ligands such as Sonic Hedgehog (Shh), Indian Hedgehog (Ihh) or Desert Hedgehog (Dhh), bind on the Patched receptor, orchestrating the nuclear localization of Gli1 proteins (Gli1-3), triggering gene expression. Hh pathway is activated in several cancers, notably in lung cancer [25]. Notably, our group and others have previously demonstrated that Shh pathway is associated with resistance to several anti-tumor therapies [26] such as chemotherapy [27,28], Tyrosine Kinase Inhibitors (TKIs) [29] and radiotherapy [30]. Like Wnt signaling, Shh pathway is also involved in the development and the activation of the immune response [31,32,33]. In gastric cancer, hedgehog signaling supports the expression of PD-L1 and tumor cell proliferation [34]. Concordantly, growing evidence from pre-clinical studies have also suggested a lower efficacy of ICIs in case of Shh pathway activation in other cancers [32,33,35]. For example, in basal cell carcinoma, where the pathway is highly active, Hedgehog signaling inhibits cytotoxic T cells via the expression of PD-1 and the recruitment of immunosuppressive regulatory T cells [31].

Overall, Wnt and Hedgehog signaling is at least in part responsible for immune evasion and resistance to ICIs, thus their expression levels could be related to the patient outcome. The purpose of this work was to assess the association between Wnt and Shh pathways activation and resistance to ICIs in a cohort of patients with advanced NSCLC. 

## 2. Results

### 2.1. Clinical Characteristics

Sixty-three (63) consecutive patients with at least one plasma sample at the introduction of the treatment and/or during the follow-up, were included between January 2015 and August 2018: 52 (82.5%) samples were collected before the introduction of the immunotherapy, 41 (65.0%) samples were collected at the first clinical evaluation (2 months after the treatment introduction). Thirty-six patients (36) (66.7%) had a corresponding tumor sample with enough tumor cells available for Gli1 and beta-catenin immunohistochemistry (IHC).

The characteristics of the patients are summarized in Table 1. Patients were mainly men (55.5%), smokers or ex-smokers (90.4%). The main histological type was adenocarcinoma (68.2%). ICI was introduced as first-line therapy in 28.6% (*n* = 18), and as second-line therapy or more in 71.4% (*n* = 45). 

The median follow-up was 13.7 months (Interquartile range, IQR 5.6–28.7) from the introduction of the ICI. The median overall survival (OS) was 16.9 months (IQR 5.9–50.3) and median progression-free survival (PFS) was 3.4 months (IQR 1.6–10.5). For patients treated with ICI as second-line therapy or more, the median OS and the median PFS were 13.7 months (IQR 5.5–35.6) and 2.8 months (IQR 1.6–7.8) respectively. For patients treated with ICI as first-line therapy, the median OS was not reached (NR) (IQR 15.3- NR) while the median PFS was 9.2 months (IQR 2.2–23).

Objective response rate (ORR) for the whole population was 36.5% (*n* = 23/63). ORR was 33.3% (*n* = 15/45) for patients treated in second line or more, and 44.4% (*n* = 8/18) for patients receiving ICI as first line. Among all patients included (*n* = 63), 25 patients (39.7%) had a primary resistance; 20 (44.4%) among patients treated in second line or more (*n* = 45) and 5 (27.8%) among patients treated in first line (*n* = 18).

### 2.2. Hh Pathway Expression and Activation

#### 2.2.1. Gli1 Expression

Among all patients included (*n* = 63), expression of Gli1 in IHC was evaluated in 36 patients (57.1%) with enough tumor tissue available. The characteristics of patients with available Gli1 expression were comparable to the global population (Supplementary Table S1). Among them, 15 (41.7%) were treated in first line and 21 (58.3%) in second line or more. ORR was 36.1 % (*n* = 13), and 13 patients (36.1%) had primary resistance to ICIs. 

**Figure 1:**
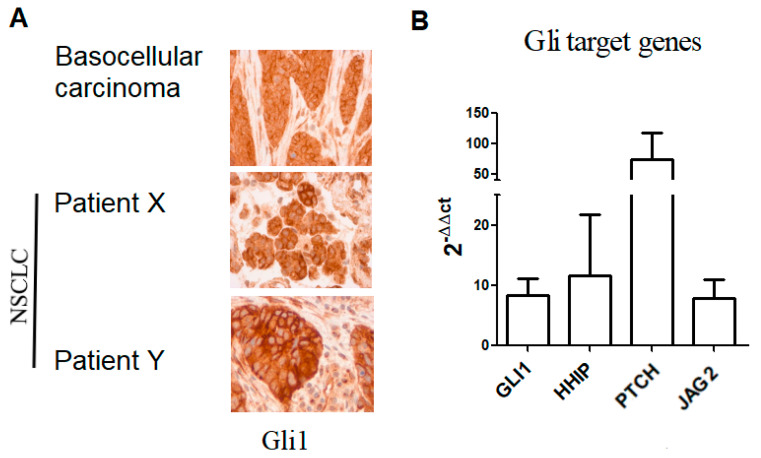
Hedgehog activation in NSCLC samples. (**A**) Immunohistochemistry analysis of Gli1 in NSCLC, patients X and Y are representative of Gli1+ patients (18/36). (**B**) mRNA expression of Gli1 target genes in NSCLC, data are expressed as mean ± SEM of 12 patients analyzed in duplicate. Two lung tissue samples not expressing Gli1 as assessed by IHC were used as reference samples or calibrators. A sample from basocellular carcinoma were used as positive control both for the IHC and the RT-qPCR. NSCLC: non-small-cell lung cancer.

**Figure 2:**
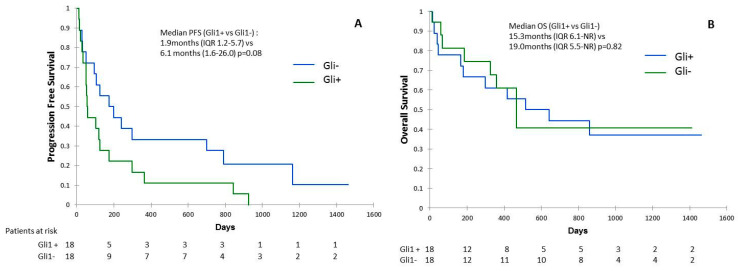
Progression-free survival (PFS) (**A**) and overall survival (OS) (**B**) according to Gli1 expression in immunohistochemistry.

We observed that 50% (*n* = 18/36) of samples analyzed were positive for nuclear Gli1 expression (Gli1+) (Figure 1A). To validate the activation of Hh signaling, we analyzed mRNA levels of Gli1 target genes including GLI1, HHIP (Hedgehog-interacting protein), *PTCH* and *JAG2* (Table S4). Consistently, we found the transcript of HHIP, *PTCH*, and *JAG2* in patients’ biopsies (Figure 1B; Figure S1A,B). ORR was lower in Gli1+ compared to patients with Gli1 negative (Gli1-) NSCLC: 20.0% vs. 55.6%, respectively (*p* = 0.015). In Gli1+ NSCLC, rate of primary resistance was 69.8%, versus 31.2% in Gli1- NSCLC (*p* = 0.04). Median PFS was shorter in Gli1+ NSCLC compared to Gli1- NSCLC: 1.9 months (IQR 1.2–5.7) vs. 6.1 months (1.6–26.0), respectively (*p* = 0.08) (Figure 2A). No difference was observed in terms of OS between the two groups: 15.3 months (IQR 6.1-NR) vs. 19.0 months (IQR 5.5-NR), respectively (*p* = 0.82) (Figure 2B).

#### 2.2.2. Plasma Shh Concentrations

Results of Shh concentrations related to outcome (ORR, primary resistance, PFS, OS) are presented in Table 2, Supplementary Tables S2 and S3. 

The median Plasma Shh concentration at baseline (*n* = 52) and at the first clinical evaluation (*n* = 41) were, respectively, 323.9 pg/mL (IQR 175.6–447.1) and 344.8 pg/mL (IQR 169.6–516.5). Tumor evaluation showed that patients with a good tumor response had higher Shh concentrations compared to patients with no tumor response: 467.4 pg/mL (IQR 282.6–693.2) vs. 268.2 pg/mL (IQR 142.25–461.3), respectively (*p* = 0.01). Patients displaying primary resistance had lower Shh concentrations compared to patients without primary resistance at the introduction of the ICI and at the first evaluation: 274.8 pg/mL (IQR 139.6–372.1) vs. 386.0 pg/mL (IQR 212.9–532.5) (*p* = 0.03) and 242.2 pg/mL (IQR 147.4–304.5) vs. 454.7 pg/mL (IQR 236.1–640.2) (*p* = 0.009), respectively. Patients with high level of Shh at first evaluation (>344.8 pg/mL) had longer OS than patients with low level of Shh (<344.8 pg/mL): 35.6 months (IQR 13.7-NR) vs. 16.2 months (IQR 5.9–34.6) respectively (*p* = 0.03). No difference was observed in terms of PFS according to Shh concentrations at first evaluation: 7.9 months (IQR 4.1–23.0) vs. 3.4 months (1.6–16.2) respectively, (*p* = 0.15) (Supplementary Table S2).

### 2.3. Wnt-Beta Catenin Pathway Expression

#### 2.3.1. Beta-Catenin Expression

Beta-catenin expression was evaluated by IHC in 36 patients (63.7%) with enough tumor tissue available. Among them, 15 (41.7%) were treated in first line and 21 (58.3%) in second line or more. The ORR was 36.1 % (*n* = 13), and 13 patients (36.1%) presenting a primary resistance to ICIs. We observed a weak nuclear staining of beta catenin in tumor cells from any tumor sample collected at diagnosis (Figure S1C). 

#### 2.3.2. Wnt1 Plasma Concentrations

Results of Wnt1 concentrations related to clinical outcomes (ORR, primary resistance, PFS, OS) are presented in Table 2, Supplementary Tables S2 and S3.

Wnt1 concentrations at the initiation (*n* = 52) of the ICI and at the first clinical evaluation (*n* = 42) were 420.9 pg/mL (IQR 282.9–693.9) and 405.6 pg/mL (IQR 295.7–625.8), respectively. 

**Figure 3:**
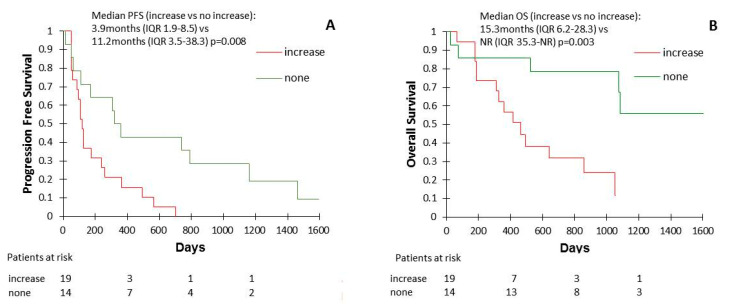
Progression-free survival (PFS) (**A**) and overall survival (OS) (**B**) according to the evolution of plasma Wnt1 concentrations during ICI treatment.

We found no correlation and/or association between Wnt1 expression levels at baseline and response rate (42.1% vs. 57.1% in other patients, *p* = 0.39) or primary resistance rate (31.6% vs. 14.3% in other patients, *p* = 0.25). However, some patient’s samples showed an increase of Wnt1 levels between the ICI initiation and the first clinical (Supplementary Table S2). These patients with increasing Wnt1 concentration had shorter PFS (median 3.9 months (IQR 1.9–8.5) vs. 11.2 months (IQR 3.5–38.3); *p* = 0.008) and OS (median 15.3 months (IQR 6.2–28.3) vs. not reached (IQR 35.3-NR); *p* = 0.003) than the other patients (Figure 3).

#### 2.3.3. Wnt2 Plasma Concentrations

Relation between Wnt2 concentrations and patient outcomes (ORR, primary resistance, PFS, OS) are presented in Table 2, Supplementary Tables S2 and S3.

Among all patients with available plasma sample at the initiation of the ICI (*n* = 49), median plasma Wnt2 concentration was 804.4 pg/mL (IQR 505.0–1671.9). Among all patients with available plasma sample at the first evaluation (*n* = 44), median plasma Wnt2 concentration was 915.4 pg/mL (IQR 488.8–1221.8). 

**Figure 4:**
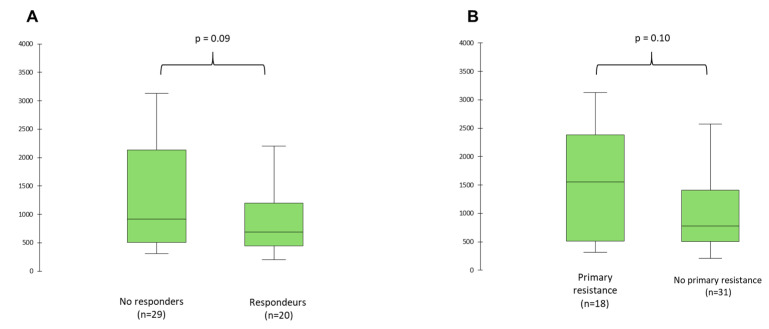
Wnt2 concentrations at baseline according to tumor response (**A**) and primary resistance (**B**).

Considering patients according to the treatment response, baseline median Wnt2 concentrations was 918.6 pg/mL (IQR 508.0–2138.4) in non-responders, vs. 689.2 pg/mL (IQR 446.0–1201.5) in responders (*p* = 0.09) (Figure 4A), and 1551.4 pg/mL (IQR 513.8–2381.2) in patients with primary resistance vs. 778.1 pg/mL (IQR 502.2–1410.9) in patients without primary resistance (*p* = 0.10) (Figure 4B). 

#### 2.3.4. Wnt3 Plasma Concentrations

Results of Wnt3 concentrations related to outcome (ORR, primary resistance, PFS, OS) are presented in Table 2, Supplementary Tables S2 and S3. 

Among all patients with available plasma sample at the initiation of the ICI (*n* = 52), median plasma Wnt3 concentration was 319.6 pg/mL (IQR 179.4–403.4). Among all patients with available plasma sample at the first evaluation (*n* = 43), median plasma Wnt3 concentration was 341.1 pg/mL (IQR 91.5–453.8). 

No significant result was observed for ORR, primary resistance, PFS and OS according to Wnt3 concentrations. 

## 3. Discussion

In our study, we found that baseline activation of Shh pathway and increase of Wnt1 during ICI treatment were associated with poor outcome in NSCLC patients treated with ICIs. These findings suggest that Shh and Wnt molecules could stand as new prognostic biomarkers for NSCLC patients treated with ICIs.

The broad role of Shh and Wnt signaling in immune response is due at least in part to the existence of a crosstalk with immune checkpoint pathways. Hh and canonical or non-canonical Wnt signaling induce the expression and activation of immune checkpoint molecules such as PD-1/PDL-1 and CTLA-4 on CD8+ T cells. Thus, studies suggest that Shh and Wnt pathways support resistance to ICIs by maintaining the expression and activation of immune checkpoint molecules [31,34,36].

In this study, Hh pathway activation, as assessed by Gli1 nuclear expression in tumoral cells, was associated with poor response to ICI, lower ORR and higher proportion of patients with primary resistance. These findings were translated into lower median PFS, without statistical significance (*p* = 0.08), possibly due to the size of the cohort. As aforementioned, there are two other Hh ligands, including Ihh and Dhh, that can induce the pathway activation [37]. Oncogenic functions for Ihh and Dhh have been described in several cancer types including hematological malignancies and solid cancers, where they have shown a biomarker value [38,39]. Even though few studies addressed the role of Ihh and Dhh in NSCLC, our data cannot exclude the involvement of the other ligands in the activation of Hh/Gli axis observed in NSCLC. However, the current result is online with previous studies, where we demonstrated that the signaling axis involving Shh and Gli1/Gli2 is associated with chemoresistance in NSCLC [28]. These results are consistent with previous studies demonstrating a role for Shh signaling in resistance to many anti-tumor therapies in NSCLC [25,26,27,28,29,30]. Concerning ICI, preclinical data suggested a low efficacy of ICI in case of Shh pathway activation [40]. Moreover, Hanna et al. observed a shift from an immune suppressive microenvironment to a pro-inflammatory immunogenic phenotype in a mice model of breast tumor treated with vismodegib, a Shh pathway inhibitor [33]. Furthermore, Shh inhibition significantly upregulated the infiltration of cytotoxic CD8 T-cells [33]. Here, we observed a positive association between Shh plasma concentrations and response to the ICI, PFS and OS. These observations could be explained either by the secretion of Shh by non-tumoral cells, or by the existence of a non-canonical Shh-independent activation of Shh pathway [41]. Further studies are needed to clarify the role of circulating Shh protein during ICI treatment.

Concerning the Wnt pathway, we did not observe any nuclear staining of beta-catenin in tumor cells at baseline, suggesting an absence of Wnt-beta catenin pathway activation in NSCLC at diagnosis. Activation of the Wnt pathway was reported in a small fraction of NSCLC patients [19], but the small number of patients in our cohort limits the interpretation of this result. However, we showed that an increase of Wnt1 concentration between the initiation of the treatment and the first evaluation was significantly associated with poor PFS and OS under ICI treatment. These results suggest that the activation of Wnt pathway in NSCLC during ICI treatment could lead to resistance to ICI. We showed recently that secondary late progression of patients treated with ICIs was associated with the acquisition of somatic mutations on Wnt pathway-related genes [24], thus reinforcing the hypothesis of the implication of Wnt pathway in secondary resistance. IHC analyses of beta-catenin on rebiopsy at the time of progression upon ICIs treatment may be able to confirm this hypothesis. A prospective trial of systematic rebiopsy at progression in lung cancer patients treated by ICI is ongoing (NCT04300062), and beta-catenin staining is notably planned on these samples. 

The main limitation of our study is the small number of available samples, inducing a lack of power for several analyses. Despite this, we were able to find significant results concerning Gli1 expression, plasma Wnt1 concentrations and poor outcome. 

## 4. Materials and Methods 

### 4.1. Patients 

All consecutive patients (*n* = 63) with advanced (not irradiable stage IIIb, or stage IV) NSCLC treated in the Department of Respiratory Medicine and Thoracic Oncology (APHP—Ambroise Pare Hospital) with pembrolizumab (first-line setting in case of IHC PD-L1 ≥ 50%, or in ≥2nd line setting in case of IHC PD-L1 ≥ 1%), or with nivolumab (≥2nd line setting), between January 2015 and August 2018 were included.

Demographic and histo-molecular data were retrospectively collected. Tumor response was assessed locally, using iRECIST criteria. Best overall response rate (ORR), progression-free survival (PFS) and overall survival (OS) were collected with a cutoff point on 07/07/2020.

### 4.2. Plasma and Tumor Tissues

Plasmas were prospectively collected and stored in the Biological Resource Center (CRB, APHP—Ambroise Pare Hospital). Briefly, two 10 mL-EDTA tubes of peripheral blood were taken at the beginning of ICI treatment and every 2 months during treatment, and plasma was isolated within one hour by centrifugating samples for 10 min at 3000× *g*, supernatant are collected and immediately conserved at −80 °C.

Available corresponding diagnostic tumor tissues (formalin-fixed paraffin-embedded blocks) were retrieved. For further IHC analyses, the number of tumor cells had to be greater than 100 per sample.

### 4.3. ELISA

Plasma concentrations of Wnt1, Wnt2, Wnt3 and Shh proteins were evaluated by ELISA [42,43,44] at ICI introduction and at the first clinical evaluation (2 months after treatment introduction). We used commercially available ELISA kits (CSB-EL026128HU (Wnt1), CSB-EL026133HU (Wnt2), CSB-EL026135HU (Wnt3), Cusabio (Houston, TX, USA); ab100639 (Shh), Abcam, Cambridge, UK), according to the manufacturer’s instructions. Optical densities were determined with spectrophotometer (Bio-Rad PR3100) measuring optical density at 450 nm. Each sample was tested in duplicate. Concentrations for each protein were calculated using standard controls (recombinant proteins) and standard curves. 

### 4.4. Immunohistochemistry (IHC)

Baseline Shh pathway activation was assessed by Gli1 nuclear expression in tumor cells (anti-Gli1 mouse monoclonal antibody sc-515751; 1:10 dilution; Santa Cruz Biotechnology, Dallas, TX, USA) and the expression of Gli1 target genes BCL-2 (anti-Bcl-2 mouse monoclonal antibody M0887; 1:30 dilution; Agilent Dako, Santa Clara, CA, USA) and Cycline D1 (anti-Cycline D1 rabbit monoclonal antibody RM9104; ready to use; Thermo Scientific, Saclay, France). Gli1, Bcl-2 and Cyclin D1 positive staining was evaluated on tumor cells only, surrounding healthy cells. Protein expression was considered as positive if more than 25% of tumor cells had a positive staining, as previously published [28]. Samples of basocellular human carcinoma [45] were used as positive control and nonspecific immunoglobulin isotype as a negative control.

Baseline Wnt pathway activation was assessed by nuclear expression of Betacatenin in tumor cells (anti-betacatenin mouse monoclonal antibody (clone 10H8); Calbiochem/Oncogene, Fontenay-sous-Bois, France). Betacatenin expression was considered as positive if ≥5% of tumor cells had a nuclear staining. Samples of human desmoid tumor [46] were used as positive control and nonspecific immunoglobulin isotype as a negative control. 

All IHC were performed on a LEICA BOND-III automate. Slides were read in blinded by a thoracic pathologist (CJ). Percentage of positive cells were generated by cell counting.

### 4.5. Reverse Transcription and Quantitative Polymerase Chain Reaction

RNA was extracted from tumor tissue using the AS1480 Maxwell RSC Simply RNA tissue kits (Promega, Madison, WI, USA) according to the manufacturer’s instructions. RNA concentrations were calculated using Multiscan GO reader, V.1.01.10 (ThermoFisher Scientific, Saclay, France). One μg RNA was reverse-transcribed in cDNA using the high-capacity RNA-cDNA kit (ThermoFisher Scientific, Saclay, France) according to the manufacturer’s instructions. cDNA amplification was performed using the TaqMan Universal PCR Master Mix, No AmpErase UNG (ThermoFisher Scientific, Saclay, France) according to the manufacturer’s instructions. TaqMan PCR primers and probes specific for Gli1 target genes and controls β-actin were purchased commercially and used as per the manufacturer’s instructions (ThermoFisher Scientific, catalog number 4331182) (Table S4). All values were normalized to the control gene β-actin using the ΔΔ Ct method. Two samples not expressing Gli1 as assessed by IHC were taken as reference samples or calibrators.

### 4.6. Ethical Considerations

A consent form was signed by all patients to collect peripheral blood samples and tissues biopsies. Plasma samples were conserved at Hospital Ambroise Paré Biological Resources Center (AFNOR NF-96900 certification). The protocol was approved by the Institutional Review Board CPP IDF n°8.

### 4.7. Statistical Analysis

ORR, PFS and OS were assessed according to plasma concentrations of Wnt1, Wnt2, Wnt3 and Shh at the initiation of ICI and at the first clinical evaluation, and the nuclear expression of Gli1 and Beta-catenin in IHC at the diagnostic. Plasma expression of Shh, Wnt1, Wnt2, Wnt3 was considered high when their expression value was above the median expression value, and low when their expression value was below the median expression value. We considered a 5% or more increase of the plasma concentration between the initiation of the treatment and the first clinical assessment as a significant increase. 

ORR was defined as the proportion of patients who achieve a complete or partial response according to iRECIST criteria, defining responders. Patients with stability or tumor progression were defined as non-responders. Patients who had tumor progression as best tumor response were considered as having primary resistance to ICI. ORR according to plasma concentration values was determined with the Mann–Whitney test. ORR according to the concentration variation and to the nuclear expression of Gli1 and Beta-catenin was determined with a Chi-square test.

OS was defined as the time from the treatment initiation to the date of death. PFS was defined as the time from the treatment initiation to the date of progression, second cancer or death. PFS and OS were assessed by the Kaplan–Meyer method. Comparison between survival curves was performed using log-rank method.

All the statistical analyses were calculated with XLStat version 21.2.3 (Addinsoft, Paris, France). *p*-value less than 0.05 was considered as significant.

## 5. Conclusions

In conclusion, this is the first study to our knowledge to find an association between the Shh and Wnt pathways and resistance to ICI in advanced NSCLC patients. These results, although preliminary, could have direct clinical implications, as drugs targeting Wnt and Shh pathways are currently available or in clinical development. These results need, however, to be confirmed in larger prospective cohorts, and further analyses in patients treated with ICI—chemotherapy combination are awaited. 

## Figures and Tables

**Table 1:** Characteristics of the patients.

Characteristics	Overall Population *n* = 63
**Age at the introduction of ICI (year-old)**	
Median	68
Range	62.0–73.5
**Gender**	
Female	28 (44.5%)
Male	**35 (55.5%)**
**Smoking status**	
Current	18 (28.6 %)
Former	39 (61.9 %)
Never	6 (9.5 %)
**Histology**	
Non squamous	43 (68.2 %)
Squamous	12 (19.0 %)
Other	8 (12.7 %)
**Molecular alteration at the diagnostic**	
*KRAS* mutation	22 (34.9 %)
*BRAF* mutation	4 (6.3 %)
*EGFR* mutation	2 (3.2 %)
*MET* amplification	1 (1.6 %)
No alteration	34 (54.0 %)
**ECOG performance-status at the introduction of ICI**	
0–1	44 (69.8 %)
2	19 (30.2 %)
**Type of ICI and number of lines before ICI**	
Pembrolizumab	20 (31.7 %)
First line	18 (28.6 %)
Second line and more	2 (3.2 %)
Nivolumab	43 (68.3 %)
First line	0 (0 %)
Second line and more	43 (68.3 %)

Data are expressed as *n* (%), although otherwise specified.

**Table 2:** Tumor response and primary resistance according to of Shh, Wnt1, Wnt2, Wnt3 plasma concentrations at ICI introduction and at the first tumor evaluation.

		Tumor Response			Primary Resistance		
		Responders (pg/mL)	Non-Responders (pg/mL)	*p*	Yes (pg/mL)	No (pg/mL)	*p*
**Shh**	Introduction	365.5 (IQR 282.3–507.6)	286.4 (IQR 142.2–434.6)	0.11	274.8 (IQR 139.6–372.1)	386.0 (IQR 212.9–532.5)	0.03
	First evaluation	467.4 (IQR 282.6–693.2)	268.2 (IQR 142.3–461.3)	0.01	242.2 (IQR 147.4–304.5)	454.7 (IQR 236.1–640.2)	0.009
**Wnt1**	Introduction	444.5 (IQR 290.6–727.4)	396.1 (IQR 282.0–668.4)	0.62	557.1 (IQR 296.3–707.1)	397,3 (IQR 260.4–560.3)	0.23
	First evaluation	503.3 (IQR 367.8–688.6)	346.6 (IQR 261.9–567.5)	0.07	371.4 (IQR 266.3–540.8)	424.4 (IQR 330.0–681.3)	0.30
**Wnt2**	Introduction	689.2 (IQR 446.0–1201.5)	918.6 (IQR 508.0–2138.4)	0.09	1551.4 (IQR 513.8–2381.2)	778.1 (IQR 502.2–1410.9)	0.10
	First evaluation	937.8 (IQR 469.4–1098.7)	835.2 (IQR 563.3–1530.5)	0.71	831.2 (IQR 534.1–1773.3)	928.6 (IQR 482.4–1131.8)	0.90
**Wnt3**	Introduction	270.4 (IQR 95.7–382.8)	331.6 (IQR 244.6–409.7)	0.20	320.6 (IQR 234.7–487.1)	318.7 (IQR155.4–386.5)	0.67
	First evaluation	358.8 (IQR 189.5–398.0)	319.2 (IQR 59.5–474.0)	0.78	335.8 (IQR 64.5–441.7)	354.6 (IQR 140.9–449.4)	0.63

IQR: Interquartile Range; NR: not reached.

## Data Availability

Data available on reasonable request.

## Supplementary Materials

The following are available online at https://www.mdpi.com/2072-6694/13/5/1107/s1. Supplementary Table S1: Characteristics of patients included with available Gli1 expression; Supplementary Table S2: OS and PFS according to plasmatic concentrations of Wnt1, Wnt2, Wnt3, or Shh at introduction of the ICI or at the first evaluation; Supplementary Table S3: Tumor response and primary resistance according to the presence of an increase of Shh, Wnt1, Wnt2, Wnt3 concentrations between the introduction of the ICI and the first evaluation. Table S4. List of probes used to analyze relative expression of Gli1 target genes. All primers were commercially acquired from ThermoFisher Scientific (France); Figure S1. (A) Shh/Gli1 activation in NSCLC samples as assessed by Immunohistochemistry analysis of Gli1 and Gli1 target genes Cyclin D1 and BCL-2. Patients X and Y are representative of Gli1+ patients (18/36); the Basocellular sample was used as positive control. (B) mRNA expression of Gli1 target genes in NSCLC, data are expressed as mean ± SEM of 7 patients analyzed in duplicate. (C) Beta-catenin expression in NSCLC as assessed by Immunohistochemistry. Patient z is representative of the whole population (n = 36). The Intra-abdominal desmoid tumor is used as positive control.

## Abbreviations

CTLA-4	cytotoxic T-lymphocyte-associated protein 4
CSCs	Cancer stem cells
ECOG	Eastern Cooperative Oncology Group
ICIs	Immune checkpoint inhibitors
IHC	Immunohistochemistry
IQR	Interquartile range
NR	Not reached
NSCLC	Non-small-cell lung cancer
ORR	Objective response rate
OS	Overall survival
PCR	Polimerase chain reaction
PFS	Progression free survival
PD-1	Programmed death-1
PD-L1	Programmed death-ligand 1
Shh	Sonic Hedgehog
