# Supplementary material for: Baseline Hedgehog Pathway Activation and Increase of Plasma Wnt1 Protein Are Associated with Resistance to Immune Checkpoint Inhibitors in Advanced Non-Small-Cell Lung Cancer
Source: Cancers (Basel). 2021 Mar 5;13(5):1107. doi: 10.3390/cancers13051107 (PMC7962040; doi:10.3390/cancers13051107)
Supplement: Supplementary file 1 [file cancers-13-01107-s001.pdf]

cancers MDPI

Table S1. Characteristics of patients included with available Gli1 expression.

Characteristics	Population with available Gli1 expression n= 36
**Age at the introduction of ICI (year-old)**	
Median	67.5
Range	62.5-72.2
**Female sex**	17 (47.2%)
**Smoking status**	
Current	11 (30.5%)
Former	22 (61 .1%)
Never	3 (8.3%)
**Histology**	
Non squamous	24 (66.7%)
Squamous	7 (19.4%)
Other	5 (13.9%)
**Molecular alteration at the diagnostic**	
KRAS mutation	12 (33.3%)
BRAF mutation	2 (5.5%)
EGFR mutation	1 (2.8%)
MET amplification	0 (0.0%)
No alteration	21 (58.3%)
**ECOG performance-status at the introduction of ICI**	
0-1	23 (63.9%)
2	13 (36.1%)
**Type of ICI and number of lines before ICI**	
Pembrolizumab	12 (33.3%)
- First line	12 (33.3%)
- Second line and more	0 (0.0%)
Nivolumab	24 (66.7%)
- First line	0 (0)
- Second line and more	24 (66.7%)

Data are expressed as n (%), although otherwise specified

Table S2. OS and PFS according to plasmatic concentrations of Wnt1, Wnt2, Wnt3, or Shh at introduction of the ICI or at the first evaluation.

		OS (months)			PFS (months)		
		**High expression**	**Low expression**	**p**	**High expression**	**Low expression**	**p**
**Shh**	Introduction	23.2 (IQR 6.1-50.3)	15.3 (IQR 5.9-35.6)	0.45	3.7 (IQR 1.8-10.5)	3.4 (IQR 1.0-9.8)	0.14
	First evaluation	35.6 (IQR 13.7-NR	16.2 (IQR 5.9-34.6)	0.03	7.9 (IQR 4.1-23.0)	3.4 (1.6-16.2)	0.15
**Wnt1**	Introduction	35.3 (IQR 6.1-50.3)	15.6 (IQR 6.2-28.3)	0.11	1.7 (IQR1.1-11.9)	3.8 (2.8-8.5)	0.69
	First evaluation	28.4 (IQR 6.1-NR)	19.1 (7.5-NR)	0.92	2.8 (IQR 1.6-11.9)	2.1 (IQR 1.6-3.9)	0.36
**Wnt2**	Introduction	21.2	15.3	0.96	3.4	5.5	0.70

		(IQR 4.5-50.3)	(IQR 9.8-35.6)		(IQR1.6-9.8)	(IQR 1.6-11.9)	
	First evaluation	28.3 (IQR 10.2-35.3)	16.2 (IQR5.8-NR)	0.99	4.9 (IQR 3.1-24.2)	4.5 (IQR 1.6-10.5)	0.08
**Wnt3**	Introduction	21.2 (IQR 5.9-NR)	15.3 (IQR 6.2-50.3)	0.90	3.5 (IQR 1.6-10.5)	3.6 (IQR 1.6-9.8)	0.77
	First evaluation	28.4 (IQR 16.2-35.6)	13.7 (IQR 6.1-NR)	0.78	10.5 (IQR 3.1-24.2)	3.5 (IQR 1.7-7.9)	0.09

IQR: Interquartile Range; NR: not reached

Table S3. Tumor response and primary resistance according to the presence of an increase of Shh, Wnt1, Wnt2, Wnt3 concentrations between the introduction of the ICI and the first evaluation.

		**n**	**ORR (n; %)**	**p**	**Primary resistance (n ; %)**	**p**
Shh	increase	12	5 (41.6)	0.24	3 (27.3)	0.64
	no increase	19	12 (63.1)		4 (20.0)	
Wnt1	increase	19	8 (42.1)	0.39	6 (31.6)	0.25
	no increase	14	8 (57.1)		2 (14.3)	
Wnt2	increase	20	8 (40.0)	0.23	6 (30.0)	0.34
	no increase	13	8 (61.5)		2 (15.4)	
Wnt3	increase	19	10 (52.6)	0.71	5 (27.8)	0.6
	no increase	14	7 (50.0)		3 (20.0)	

ORR: overall response rate;

Table S4. List of probes used to analyze relative expression of Gli1 target genes. All primers were commercially acquired from ThermoFisher Scientifi (France).

**Genes**	**Taqman probes**
GLI1	Hs01110773_g1
HHIP	Hs01011015_m1
PTCH	Hs00288486_m1
JAG2	Hs00171432_m1
ACTB	Hs99999903_m1

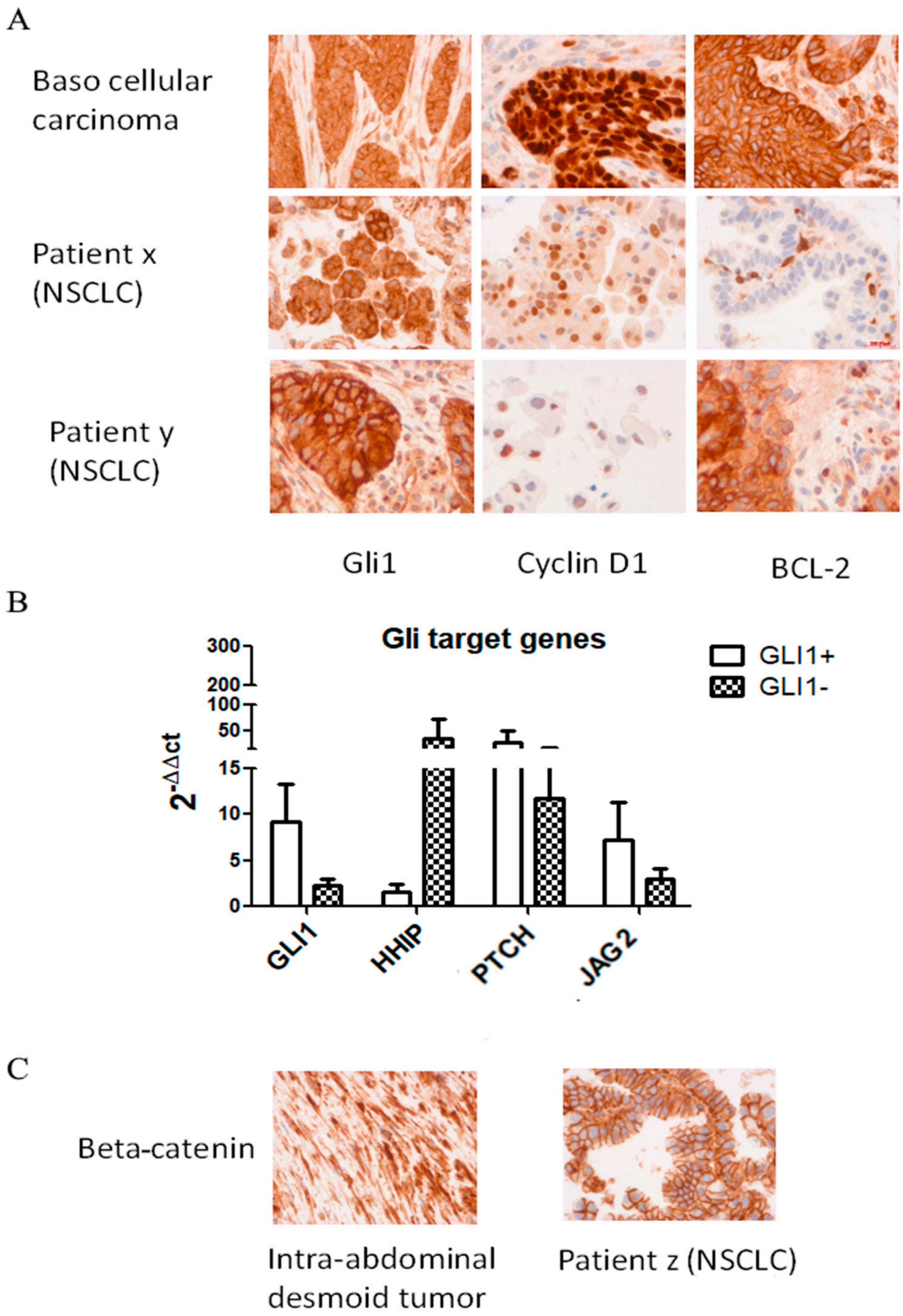

Figure S1. (**A**) Shh/Gli1 activation in NSCLC samples as assessed by Immunohistochemistry analysis of Gli1 and Gli1 target genes Cyclin D1 and BCL-2. Patients X and Y are representative of Gli1+ patients (18/36); the Basocellular sample was used as positive control. (**B**) mRNA expression of Gli1 target genes in NSCLC, data are expressed as mean ± SEM of 7 patients analyzed in duplicate. (**C**) Beta-catenin expression in NSCLC as assessed by Immunohistochemistry. Patient z is representative of the whole population (n = 36). The Intra-abdominal desmoid tumor is used as positive control.
